# Knowledge of Bat Rabies and Human Exposure Among United States Cavers

**DOI:** 10.3201/eid0805.010290

**Published:** 2002-05

**Authors:** Robert V. Gibbons, Robert C. Holman, Stephen R. Mosberg, Charles E. Rupprecht

**Affiliations:** * Centers for Disease Control and Prevention, Atlanta, Georgia, USA; † National Speleological Society, Huntsville, Alabama, USA

**Keywords:** rabies, lyssa virus, cavers, spelunkers, vaccination, bats, zoonoses

## Abstract

We surveyed cavers who attended the National Speleological Society convention in June 2000. Fifteen percent of respondents did not consider a bat bite a risk for acquiring rabies; only 20% had received preexposure prophylaxis against the disease. An under-appreciation of the risk for rabies from bat bites may explain the preponderance of human rabies viruses caused by variant strains associated with bats in the United States.

Over the past century, human rabies has become exceedingly rare in the United States. The decreasing incidence of human rabies has followed the decline of rabies in domestic dogs. From 1946 to 1965, 236 human *Rabies virus* (RABV) infections were reported in the United States. From 1946 through 1949, the number of human RABV infections averaged 24/year, declining to 1.5/year from 1962 through 1965. Ninety percent of RABV infections were caused by dog bites from 1946 through 1949, decreasing to 67% from 1962 through 1965 (*1*). As canine rabies declined, the relative importance of other reservoirs in the United States increased. From 1970 to 1989, human infections averaged 3.3/year. Of these infections, 45% were caused by canine RABV variants, 30% were caused by bat RABV variants, and one was caused by a corneal transplant from an unsuspected rabies patient; all but one was acquired outside the United States (*2*,*3*). From 1990 through 2000, bat RABV variants have emerged as the predominant cause of human rabies in the United States (*4*). In the past 11 years, total human rabies deaths have averaged 2.9/year, and 24 (75%) of 32 deaths were due to bat RABV variants. If the six cases caused by foreign canine RABV variants are excluded, then 24 (92%) of the 26 human rabies deaths acquired domestically were caused by bat RABV variants. The other two cases were due to a dog/coyote RABV variant found in Texas (*4*).

Confusion remains about potential exposures to rabies from bats. Only 2 (8%) of the 24 patients with human rabies caused by bat RABV variants had a definitive history of a bat bite. Nine patients (38%) had a history of direct physical contact with bats, 5 (21%) had a history of a bat inside the living area, and 8 (33%) had no history of proximity to bats (*4*). Because of the paucity of bat (or other animal) bite histories, could these human rabies cases have been acquired through aerosol transmission? The diagnosis of rabies in two people who had no known history of a bite, but who worked extensively in caves inhabited by bats, received considerable attention in 1953 (*1*,*2*). Although the aerosol route is considered a possible mechanism of RABV acquisition, few data support such transmission under typical field conditions. A more plausible hypothesis is that many people may not be aware that a bat bite is a risk for rabies transmission and fail to report it.

Because of the potential contact with bats, cavers are considered at a higher risk for rabies exposure than the general population. Since the 1960s, the recommendation has been that the cavers receive rabies preexposure prophylaxis (PreEP) (*5*). The objectives of this study were to learn about cavers’ knowledge of the risks for bat-to-human rabies transmission and to quantify cavers’ use of rabies PreEP prophylaxis and postexposure prophylaxis (PostEP).

## The Study

We administered a survey to cavers attending the National Speleological Society Convention in Elkins, West Virginia, USA, in June 2000. The survey was included in the convention registration packet. Verbal reminders to return the survey were given, and collection boxes were located at several sites at the convention.

The survey asked respondents about demographic information, how long and how many times they had been caving, how often they encountered bats when caving, if they had been advised to receive the rabies PreEP and if they had received it, if they considered specific scenarios (bat bite, bat scratch, bat on skin, bat on clothing, indirect contact with bats) as a potential risk for rabies, if they had ever had a potential exposure to rabies, and if they had ever received rabies PostEP.

Categorical variables were compared using the chi-square test or the Fisher’s exact test (2-tailed), as appropriate. Continuous variables were analyzed with the Wilcoxon rank-sum test (*6*). Multivariate logistic regression was used for multivariate analysis.

Questionnaires were returned from 392 (26%) of 1,508 cavers attending the convention. The respondents’ mean age was 47 (range 12-84) years, 68% were male, and 76% were college graduates. The respondents caved a mean of 23 (range 1-58) years and a mean of 16 (range 0-150) times in the past year. When asked how often they see bats on their caving trips, 1% responded never, 29% sometimes, 22% about half the time, 43% often, and 5% always. Respondents were asked to address whether specific scenarios with bat(s) were considered a risk for rabies (Table 1).

**Table 1:** The number of cavers who considered the scenario as a risk for rabies

Scenario	>College degree	No college degree	Total (%)
	(n=298)	(n=94)	
Bat bite	262/294 (89)	69/93 (74)^a^	331/387 (86)
Bat scratch	191/290 (66)	42/92 (46)^a^	233/382 (61)
Bat on skin	42/292 (14)	9/93 (10)	51/385 (13)
Bat on clothing	10/293 (3.4)	1/93 (1.1)	11/386 (2.9)
Being around bats^b^	37/293 (13)	8/93 (8.6)	45/386 (12)

^a^ For having >college degree compared to no college degree, p<0.001. ^b^ Indirect contact with bats.

The respondents who thought a bat bite was not a risk for rabies were younger (43 versus 48 years, p=0.009) and less educated (43% versus 21% were not college graduates, p=0.005) but did not differ significantly by gender, number of years caving, or number of times caving in the past year. The respondents who thought that indirect contact with bats was a risk for rabies were older (52 versus 46 years, p<0.001), and caved more years (28 versus 22; p<0.001). They did not significantly differ by gender, education, or number of times caving in the past year. Seventy-six (20%) respondents received PreEP (Table 2). In multivariate analysis, having been advised to receive the vaccine was independently associated with having received it (odd ratio = 31; 95% confidence interval 15 to 61).

**Table 2:** Number of cavers who had/had not received preexposure prophylaxis (PreEP)

Characteristic	Received (n = 76)	Not received (n = 313)	p value
College graduate	63/76 (83%)	231/311 (74%)	0.12
Advised to get PreEP^a^	56/75 (75%)	31/315 (10%)	<0.001
Male gender	55/76 (72%)	210/312 (67%)	ns
See bats > half of the time	57/71 (80%)	211/311 (68%)	0.04
Mean age	49 years	46 years	ns
Mean years caving	26 years	22 years	0.01
Mean times caving per year	34/year	13/year	< 0.001

^a^The only variable independently associated with receiving PreEP.

Eighty-eight (23%) respondents had been advised to receive PreEP. Those who caved more years (25 versus 22, p=0.05), and more times in the last year (25 versus 15, p<0.001) were more likely to have been advised to have PreEP. College graduates were more likely to be advised to have PreEP, but statistical significance was not found (24% versus 17%, p=0.14). Those advised to get PreEP did not differ by age or gender. Of the 66 respondents advised to get PreEP because of caving, 37 (57%) had done so; of the 20 advised to get PreEP for other reasons, 17 (85%) had done so. Twenty-four (1.6%) respondents felt they had been potentially exposed to rabies. Of the 24, only 5 involved exposures to bats (3 from bites), and only 1 indicated this exposure was directly associated with caving.

## Conclusions

Despite the cavers’ education level and their familiarity with bats, 14% of the cavers did not consider a bat bite risk for rabies. When only the cavers without a college degree were considered, 26% did not think a bat bite was a risk for rabies. Given the general public’s assumed education level and overall lack of familiarity with bats, the percentage of the public who do not consider a bat bite a risk for rabies is probably higher than (or closer to) 26%, than 14%. If so, this would support the hypothesis that people may lack the knowledge to seek medical care if a bat bites them. Unlike bites from larger mammalian carnivores, lesions resulting from a bat bite probably will not warrant seeking medical care. In addition, 39% of cavers did not think a bat scratch was a risk for rabies. Technically, a scratch contaminated with saliva is an exposure, but scratches alone are less likely to transmit rabies than a bite. The practical problem arises in the consideration of scratches from bats. Does the patient know if the scratch is contaminated with saliva? And more importantly, can a patient discern a scratch from a bite, particularly under the darkened and tight recesses of a cave?

Eleven percent of cavers felt that indirect contact with bats to be risk for rabies. Some cavers (especially older, more experienced members) may possess knowledge of those rare cases of human rabies that are attributed to aerosol transmission. Two infections in the 1950s, commonly attributed to aerosol transmission in crowded bat caves (in a bat researcher and a mining engineer), had other possible mechanisms of infection (*7*,*8*), and no other infections have been reported in cavers. Interestingly, the lack of rabies cases in cavers is evidence against the occurrence of aerosol transmission, except under extraordinary circumstances. The respondents in our study, if projected to only cavers who are members of the NSS, represent over 4 million caving episodes; nearly 60% involved cavers with no PreEP. Of course, the expected prevalence of rabies in freeranging bats is low, probably <1% (*9*).

This survey is limited by a low response rate and may be subject to selection bias. Those who did respond may be more or less familiar with rabies than the average caver. In addition, the survey may be subject to response bias. Relationships demonstrated are associations; cause and effect cannot be definitively determined.

Nevertheless, our study suggests that despite longstanding guidelines for cavers to receive PreEP for rabies, only 20% have done so. The increase is modest when compared to a survey conducted in 1970 of 239 cavers, which found that only 14% had received PreEP (CDC, unpub. data). Increasing the cavers’ awareness about the recommendation may increase compliance, as 64% of those advised to receive PreEP had done so, compared to 6% (n=19) of those not advised to do so. In fact, this was the only independent predictor of receiving PreEP. A future survey of the general public is indicated to explore their knowledge and attitudes towards bats, rabies, and the risk for acquisition.
